# A prospective cohort study of rituximab in the treatment of refractory nephrotic syndrome

**DOI:** 10.1007/s11255-021-02860-4

**Published:** 2021-04-27

**Authors:** Jing Xu, Ying Ding, Li Wan, Qinghua Yang, Zhen Qu

**Affiliations:** Department of Nephrology, Peking University International Hospital, Beijing, 102206 People’s Republic of China

**Keywords:** Refractory nephrotic syndrome, Rituximab, eGFR, Urine protein, Albumin

## Abstract

**Objective:**

To explore the efficacy and safety of rituximab (RTX) in the treatment of autoimmune nephropathy manifested as refractory nephrotic syndrome (RNS).

**Methods:**

A single-center prospective cohort study was conducted on RNS patients treated with RTX between March 2017 and December 2019. The subjects were divided into the primary nephropathy (PN) group and the secondary nephropathy (SN) group. Based on the estimated glomerular filtration rate (eGFR) before RTX treatment, the SN group was then divided into the SN-1 group (eGFR ≥ 30 ml/min) and the SN-2 group (eGFR < 30 ml/min). Biochemical parameters and clinical data were recorded during follow-up.

**Results:**

Fifty-four patients were followed up for at least 6 months. The overall remission rates were 65%, 66.7%, 27.3% in the PN, SN-1, and SN-2 groups, respectively (*P* = 0.022). Kaplan–Meier analysis showed a significant difference of the renal survival among the three subgroups (*P* < 0.001). Multivariate Cox regression analysis showed that eGFR value before treatment was an independent predictor (HR 0.919, 95%CI 0.863–0.979) for renal survival. In terms of adverse events, infection accounted for 56.6%. The incidence of severe infection was 10%, 25% and 50% in PN group, SN-1 group and SN-2 group, respectively.

**Conclusions:**

RTX may be a promising option in RNS patients with eGFR ≥ 30 ml/min/1.73m^2^. However, it has little effect on prognosis in patients with secondary RNS with eGFR < 30 ml/min/1.73m^2^, but with a high risk of severe infection.

## Introduction

Rituximab (RTX), a chimeric anti-CD20 monoclonal antibody, has been initially approved for the treatment of non-Hodgkin's lymphoma [1]. Subsequently, the indications of RTX as an immunosuppressive agent have extended to primary glomerular diseases such as idiopathic membranous nephropathy (IMN), minimal change disease (MCD), focal segmental glomerulosclerosis (FSGS), and nephropathy secondary to autoimmune diseases such as anti-neutrophil cytoplasmic antibodies (ANCA)-associated vasculitis (AAV), lupus nephritis (LN), thrombotic microangiopathy (TMA).

In terms of primary glomerular diseases, the efficacy and safety of RTX in the treatment of IMN have been proved. Dahan et al. [2] found a positive effect of RTX on proteinuria remission in IMN patients and proved that addition of RTX to non-immunosuppressive antiproteinuric treatment (NIAT) does not affect safety. MENTOR study [3] has found that RTX was no less than cyclosporine when it induced complete or partial remission of proteinuria at 12 months, and maintained proteinuria remission for up to 24 months during IMN treatment, and the incidence of adverse reactions was similar. GEMRITUX study [4] has reported that in the treatment of IMN, using RTX alone has similar efficacy and fewer side effects compared with a combination of glucocorticoids and cyclophosphamide. RTX is also effective and safe in the treatment of refractory MCD and FSGS in children [5, 6]. For adults with MCD and FSGS, retrospective or observational studies have shown that RTX can reduce the recurrence of nephrotic syndrome and dosage of steroid and immunosuppressant [7–10]. Meanwhile, in terms of nephropathy secondary to autoimmune diseases, although Rovin's study [11] has shown no statistical difference in remission rate between RTX and placebo in LN patients, some case reports or case series have pointed out that RTX may be beneficial to LN refractory to previous immunosuppressive therapy [12–16]. Post-hoc analyses of Jones's study [17] and Stone’s study [18] have suggested that the efficacy and safety of RTX are non-inferior to standard Cyc-Aza in AAV patients with kidney involvement. Particularly, in the study of Stone, RTX-treated group has higher remission rate than that in the control group in recurrent AAV cases.

Refractory nephrotic syndrome (RNS) is characterized by insensitivity to or dependence on glucocorticoids and immunosuppressants, and repeated recurrence, which is a difficult clinical problem. Meanwhile, among patients with secondary RNS, many suffer from protracted and recurrent stage 3–4 chronic kidney disease (CKD), but there is no study reporting the efficacy of RTX in this kind of patients. In view of the beneficial effects of RTX in the treatment of patients with primary or secondary RNS, we designed this single-center prospective cohort study to observe the efficacy and safety of RTX in autoimmune kidney diseases manifested as RNS.

## Patients and methods

### Study subjects and grouping

A total of 60 patients with nephrotic syndrome (NS) who visited the Department of Nephrology of Peking University International Hospital between March 2017 and December 2019 and were planned to receive RTX were selected. The inclusion criteria were as follows: (1) with clinical evidence of RNS, that is, NS characterized by glucocorticoids resistance, glucocorticoids dependence, or repeated recurrence; (2) with a definite renal pathological diagnosis; (3) age ≥ 18 years. The exclusion criteria were as follows: (1) with following secondary causes of NC: viral hepatitis, tumors, infections, medications, diabetes; (2) with reduced kidney volume (long diameter of either side < 9.0 cm) confirmed by B-mode ultrasound; (3) with complications including tumors, hematopathy, chronic wasting disease, pregnancy, and absence of limbs; (4) with previous application of RTX. This study was performed with approval from the ethics committee of our hospital and with informed consent from each patient.

According to the diagnosis of primary diseases, the subjects were divided into the primary nephropathy group (PN group) and secondary nephropathy group (SN group). The SN group was then divided into two subgroups based on the estimated glomerular filtration rate (eGFR) level before RTX treatment, namely SN-1 group (eGFR ≥ 30 ml/min) and SN-2 group (eGFR < 30 ml/min). Both SN and PN are autoimmune diseases.

A total of three TMA patients with complement-mediated hemolytic uremic syndrome were included in our study, 1 in the SN-1 group and 2 in the SN-2 group. These three patients had anti-complement factor H autoantibodies. In addition to intravascular hemolysis, all three patients had acute kidney injury combined with hypertension and nephrotic-range proteinuria, which met the diagnostic criteria for NC. They responded to plasma exchange therapy but still relapsed repeatedly, and a combination of steroid and immunosuppressants (CYC, MMF) was not effective. Therefore, they were then treated with RTX. In the SN-2 group, both cases had complications of pulmonary infection; one patient had reduced urine protein (Upro) but with progressive decline of renal function, who entered the stage of regular dialysis, while the other had stable renal function. In the SN-1 group, the patient had stable condition after treatment and urine protein turned negative.

### Treatment protocols

All patients treated for the first time were admitted for observation. Their previous treatment regimens (types of glucocorticoids and immunosuppressants, medication regimens) and infectious complications occurred 6 months before admission were recorded, and clinical data were collected. After admission, their condition was reassessed. Following exclusion of existing infection, RTX treatment was given. The treatment protocol was as follows. Induction treatment: RTX was intravenously injected at a single dose of 375 mg/m^2^ body surface area (BSA) weekly for 4 weeks. Maintaining treatment: an additional single dose of 375 mg/m^2^ BSA of RTX was administrated weekly for 1 week if the peripheral CD19+B lymphocyte count was more than 5 cells/μl during the follow-up visit. All patients were followed-up for more than 6 months.

### Observation parameters

The changes of parameters were examined, including 24-h Upro, serum creatine (SCr), albumin (ALB), peripheral white blood cell (WBC), red blood cell (RBC), platelet (PLT), total count of lymphocytes, CD4+lymphocyte count and CD19+lymphocyte count. CKD-EPI equation was employed to calculate eGFR [19]. And adverse events during the follow-up period were recorded.

### Outcome measures

Efficacy evaluation based on remission state of nephropathy: (1) complete remission: Upro < 0.3 g/d, ALB > 30 g/l and renal function was normal; (2) partial remission: Upro was 0.3–3.5 g/d and decreased by more than 50% compared with that before treatment, ALB ≥ 30 g/L, and renal function was stable; (3) ineffective: Upro was decreased by less than 50% compared with that before treatment, ALB < 30 g/l, or renal function deteriorated. (4) recurrence: Upro > 3.5 g/d or > 50% of the baseline value in patients with complete and partial remission.

Safety evaluation: the primary endpoints were death, maintenance hemodialysis, or end-stage renal disease (ESRD) (eGFR < 15 ml/min lasted for more than 3 months), and the secondary endpoint was severe infection (requiring hospitalization and an anti-infective course for more than 2 weeks). All adverse events were recorded during the follow-up period.

### Statistical analysis

SPSS 20.0 (Chicago, IL, USA) was used for statistical analysis. Results were expressed as frequencies and percentages for categorical variables, mean ± standard deviation for variables with continuous normal distribution, and median (interquartile range) for variables with continuous non-normal distribution. Intergroup comparison was conducted through one-way analysis of variance (ANOVA) for samples with a normal distribution and through rank sum test for samples with a skewed distribution. Categorical data, frequency of complete remission, and frequency of recurrence were analyzed using the *χ*^2^ test. Kaplan–Meier survival analysis was performed for endpoint events, and multivariate Cox regression (method: Forward LR, with variable entry criteria *α* = 0.05, *β* = 0.10 and test level *α* = 0.05) was used to analyze the risk factors affecting endpoint events. *P* < 0.05 suggests a statistical significance.

## Results

### Follow-up results

**Fig. 1:**
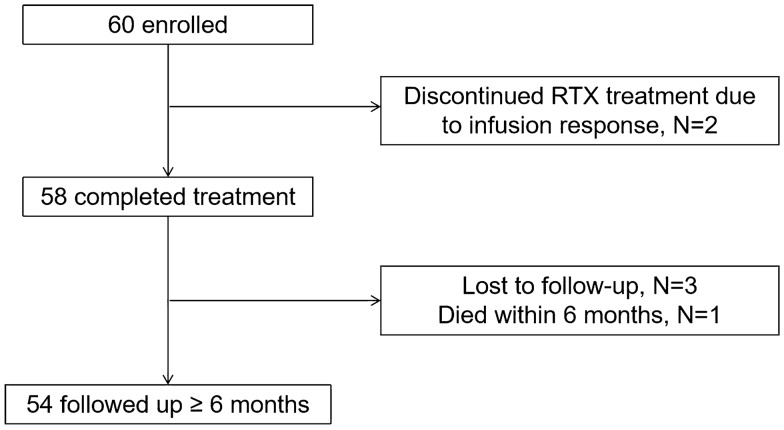
Study flow chart

Among the 60 patients, 54 patients were followed-up for more than 6 months (average 15.91 ± 9.78 months). In addition, two patients had infusion reactions to the first application of RTX and thus stopped medication three patients were lost to follow-up, and one patient died at the third month of treatment (Fig. 1).

### Baseline data

In the PN group (*n* = 20), the renal pathological types include IMN, MCD, and FSGS. In the SN-1 (*n* = 12) and SN-2 (*n* = 22) groups, the primary diseases leading to NC include systemic lupus erythematosus (SLE), AAV, and complement-mediated HUS (C-HUS) (Table 1). Among them, all IMN patients were positive for anti-PLA2R antibody, all AAV patients were positive for MPO-ANCA, and all C-HUS patients were positive for anti-factor H autoantibodies.

**Table 1:** Baseline data of the three groups of patients

Groups (cases)	PN (*N* = 20)	Comparison between SN-1 and PN	SN-1 (*N* = 12)	Comparison between SN-2 and PN	SN-2 (*N* = 22)	Comparison between SN-2 and SN-1	Overall comparison
	MN 13 (65%)		SLE 9 (75%)		SLE 15 (68.2%)		
Primary diseases *N*(%)	MCD 6 (30%)		AAV 2 (16.7%)		AAV 5 (22.7%)		
	FSGS 1 (5%)		C-HUS 1 (78.3%)		C-HUS 2 (9.1%)		
Gender (male) *N* (%)	16 (80%)	*χ*^2^ = 4.885, *P* = 0.027	5 (41.7%)	*χ*^2^ = 5.30, *P* = 0.021	10 (45.5%)	*χ*^2^ = 0.045, *P* = 0.832	*χ*^2^ = 6.677, *P* = 0.035
Age (year)	46.45 ± 17.53	–	33.08 ± 12.75	–	47.5 ± 19.28	–	*F* = 2.461, *P* = 0.099
Medical history (month)	21 (15, 31)	*H* = 9.633, *P* = 0.093	73 (9, 1117)	*H* = 12.050, *P* = 0.013	69 (11.75, 135)	*H* = 2.417, *P* = 0.668	*H* = 6.573, *P* = 0.037
Follow-up time(m)	15.75 ± 9.4	–	14.5 ± 8.6	–	16.8 ± 11.2	–	*F* = 0.215, *P* = 0.807
First round RTX dose(mg/m^2^)	1050.49 ± 291.81	–	1103.88 ± 241.18	–	947.19 ± 294.21	–	*F* = 1.093, *P* = 0.345
SCr (µmol/L)	92.50 (72.25,109.75)	*H* = 0.714, *P* = 1.000	96.50 (58.00,151.00)	*H* = 26.014, *P* < 0.001	329.5 (275.50, 438.75)	*H* = 26.727, *P* < 0.001	*H* = 37.242, *P* < 0.001
eGFR(ml/min*1.73m^2^)	78.79 ± 32.71	*P* = 0.986	83.20 ± 40.15	*P* < 0.001	15.33 ± 4.97	*P* = 0.001	*F* = 36.883, *P* < 0.001
BUN (mmol/L)	8.04 ± 4.55	*P* = 0.071	11.88 ± 5.88	*P* < 0.001	20.64 ± 5.50	*P* < 0.001	*F* = 29.115, *P* < 0.001
Upro (g/24 h)	12.59 ± 4.86	*P* = 0.008	7.14 ± 3.95	*P* < 0.001	4.83 ± 2.31	*P* = 0.258	*F* = 22.189, *P* < 0.001
ALB (g/L)	21.54 ± 5.63	*P* = 0.072	29.37 ± 9.42	*P* < 0.001	33.10 ± 595	*P* = 0.579	*F* = 15.985, *P* < 0.001
UA (µmol/L)	408.95 ± 129.86	–	459.3 ± 110.53	–	488.24 ± 15.98	–	*F* = 2.180, *P* = 0.124
WBC (10^9^/L) non-normal	9.36 ± 3.38	*P* = 0.748	9.26 ± 3.13	*P* = 0.004	6.53 ± 2.53	*P* = 0.017	*F* = 5.529, *P* = 0.007
L (10^9^/L)	1.7 (1.24, 2.78)	*H* = 4.769, *P* = 1.000	1.31 (0.98, 2.05)	*H* = 14.658, *P* = 0.004	0.95 (0.66, 1.30)	*H* = 9.889, *P* = 0.247	*H* = 10.810, *P* = 0.004
N (10^9^/L)	6.91 ± 3.35	–	7.52 ± 2.57	–	5.23 ± 2.41	–	*F* = 2.940, *P* = 0.062
RBC (10^12^/L)	4.15 ± 0.82	*P* = 0.003	3.34 ± 0.48	*P* < 0.001	3.18 ± 0.60	*P* = 0.560	*F* = 11.426, *P* < 0.001
PLT (10^9^/L)	260.05 ± 54.46	*P* = 0.806	259.70 ± 146.82	*P* < 0.001	149.10 ± 62.46	*P* = 0.002	*F* = 10.805, *P* < 0.001
CD19 + cell (cell/μL)	234.0 (116.0, 354.9)	–	108.0 (65.5, 203.0)	–	140.5 (46.25, 186.25)	–	*H* = 5.031, *P* = 0.081
CD4 + cell (cell/μL)	819.0 (523.5, 1443.0)	H = 12.026, P = 0.033	378 (275.5, 619.5)	H = 13.804, P = 0.002	325.0 (211.00, 645.50)	H = 11.778, P = 0.745	H = 10.271, P = 0.006

The laboratory indexes before and after rituximab (RTX) treatment were compared between groups. One-way analysis of variance (ANOVA) was performed for measurement data with normal distribution and equal variance, while Kruskal–Wallis non-parametric test was performed for measurement data with on non-normal distribution or unequal variance. Chi-squared test was performed for enumeration data

*SCr* serum creatinine; *eGFR* estimated glomerular filtration rate; *BUN* blood urea nitrogen; *Upro* urine protein; *ALB* albumin; *UA* uric acid; *WBC* white blood cell; *L* lymphocyte count; *N* neutrophil count; *RBC* red blood cell; *PLT* platelet

The proportion of males in the PN group was higher than that in the SN-1 group and the SN-2 group. No statistical differences were observed among the three groups in average age, average follow-up time, dosage of RTX in the first round, neutrophil level before treatment, CD19+lymphocyte count and UA level. Before treatment, the counts of RBC and CD4^+^ lymphocytes in SN-1 group and SN-2 group were lower than those in the PN group, and the counts of WBC, lymphocytes and PLT in the SN-2 group were lower than those in the SN-1 group and PN group (Table 2). In addition, all patients had been treated with glucocorticoids and more than one type of immunosuppressant prior to RTX treatment (Table 2).

**Table 2:** Previous application of immunosuppressants in three groups of patients

Types of immunosuppressants	PN (*N* = 20)	SN-1 (*N* = 12)	SN-2 (*N* = 22)
CYC	6 (30%)	9 (75%)	18 (81.8%)
CsA	14 (70%)	1 (8.3%)	3 (13.6%)
Tacrolimus	5 (25%)	3 (25%)	0
MMF	4 (20%)	6 (50%)	9 (40.9%)
AZA	0	0	1 (4.5%)
LF	0	0	6 (27.2%)

*CYC* cyclophosphamide; *CsA* cyclosporine A; *MMF* mycophenolate mofetil; *AZA* azathioprine; *LF* Leflunomide

### Therapeutic efficacy

B cell depletion and reconstitution: the peripheral blood B-cell count of all patients decreased to less than 5 cells/ul within 1 month after treatment. B-cell depletion was maintained for 7.9 ± 3.0 months in the PN group, 9.0 ± 2.6 months in the SN-1 group, and 9.6 ± 3.5 months in the SN-2 group, with no statistical difference among the three groups (*F* = 1.162, *P* = 0.323). The B-cell reconstitution occurred within 6 months in 5 cases (19.2%) in the PN group, 1 (7.1%) case in the SN-1 group, and 0 case in the SN-2 group.

**Fig. 2:**
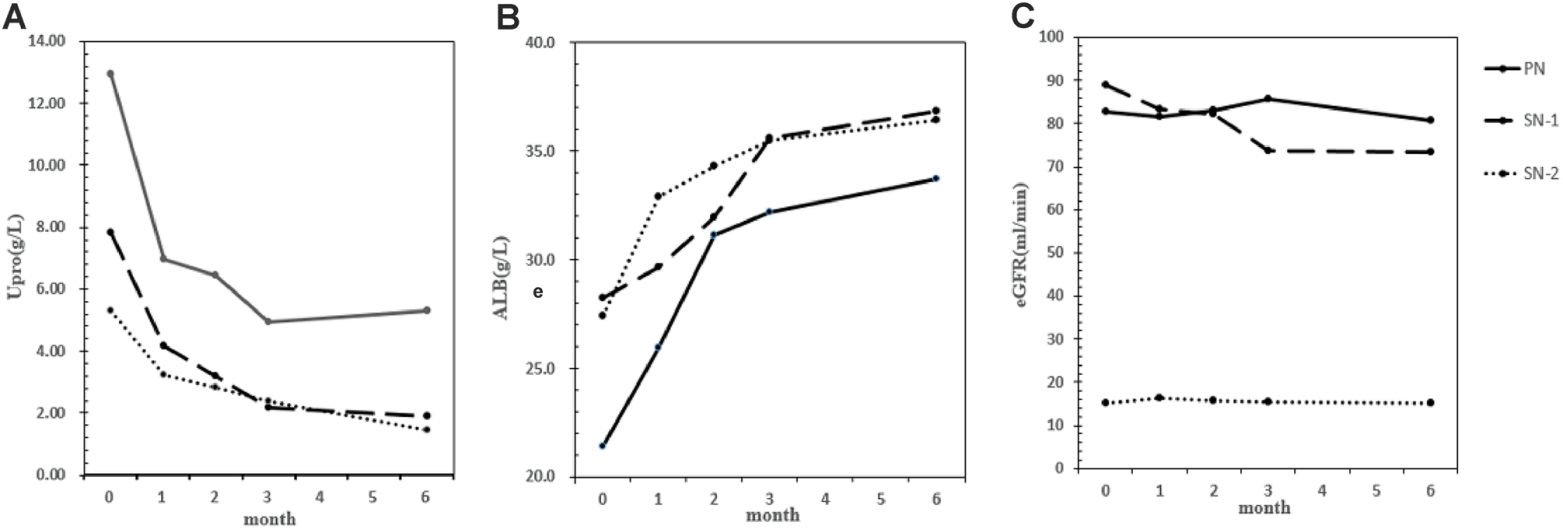
Dynamic changes of Upro, ALB and eGFR in three groups 6 months after RTX treatment

Changes in laboratory indexes before and after treatment: by repeated measures ANOVA, the general trend of nephrotic indexes after RTX treatment showed a decrease in Upro (*F* = 22.443, *P* < 0.001), an increase in ALB (*F* = 24.058, *P* < 0.001) and no significant change in eGFR levels (*F* = 2.000, *P* = 0.134). The change range of each index from baseline was similar among the three groups (Table 3, Fig. 2).

**Table 3:** Dynamic changes of laboratory indexes in three groups 6 months after rituximab treatment

Parameters	Groups	Time points					*F*	*P*
		Month 0	Month 1	Month 2	Month 3	Month 6		
Upro (g/24 h)	PN	12.93 ± 5.21	6.98 ± 5.98	6.46 ± 6.19	4.93 ± 5.47	5.29 ± 7.88	(− 6.806, − 0.130)	0.042^a^
	SN-1	7.84 ± 3.86	4.15 ± 3.01	3.20 ± 2.87	2.18 ± 1.89	1.89 ± 2.1	(− 6.895, − 1.686)	0.002^b^
	SN-2	5.29 ± 2.58	3.25 ± 2.81	2.81 ± 2.78	2.37 ± 2.08	1.43 ± 1.02	(− 4.161, 2.516)	0.621^c^
							*F* = 5.961	0.006^d^
ALB (g/L)	PN	21.4 ± 5.47	25.96 ± 8.25	31.11 ± 7.87	32.2 ± 59.65	33.72 ± 9.70	(− 2.0135, 9.181)	0.203^a^
	SN-1	28.25 ± 10.35	29.666 ± 9.55	31.95 ± 7.69	35.61 ± 6.28	36.82 ± 4.81	(0.965, 10.105)	0.019^b^
	SN-2	27.40 ± 8.67	32.88 ± 6.10	34.29 ± 5.42	35.49 ± 6.17	36.40 ± 4.98	(− 3.646, 7.549)	0.484^c^
							*F* = 3.063	0.059^d^
eGFR (ml/min)	PN	82.76 ± 32.17	81.64 ± 31.86	83.08 ± 32.46	85.54 ± 30.18	80.62 ± 31.07	(− 23.411, 18.973)	0.833^a^
	SN-1	88.93 ± 36.24	83.41 ± 34.27	82.20 ± 33.38	73.55 ± 35.37	73.45 ± 32.23	(− 84.236, − 49.803)	*P* < 0.001^b^
	SN-2	15.11 ± 4.76	16.18 ± 7.58	15.83 ± 7.65	15.32 ± 9.72	15.09 ± 11.76	(− 86.203, − 43.397)	*P* < 0.001^c^

The laboratory indexes of the three groups were measured by repeated measures analysis of variance

^a^Comparison between PN and SN-1

^b^Comparison between SN-1 and SN-2

^c^Comparison between SN-2 and PN

^d^The overall comparison

Remission: RTX treatment was effective in 27 (50%) of 54 patients, including 7 (12.96%) cases with complete remission and 20 (37.04%) cases with partial remission. In the PN, SN-1, SN-2 groups, the complete remission rates were 25%, 8.3%, 4.5%, respectively, and partial remission rates were 40%, 58.3%, and 22.7%, respectively, and the overall effective rates were 65%, 66.7%, and 27.3%, respectively (*χ*^2^ = 7.679, *P* = 0.022).

Recurrence: in the follow-up period, 4 (20%) cases in the PN group relapsed, including two IMN patients, two MCD patients. Two IMN patients relapsed 6 months after partial remission, without B-cell constitution but with a decrease of anti-PlA2R antibody at relapse. In these two patients, the glucocorticoids maintenance dose at relapse was prednisone 10 mg QD, and partial remission was achieved again 2 months after administration of a low-dose of tacrolimus. Two MCD patients relapsed 6 months and 14 months after complete remission, respectively. The glucocorticoids dose for them was prednisone 10 mg QD and 15 mg QD, respectively, at relapse. In these two patients, after peripheral blood B-cell reconstitution was detected, the second round of RTX with a single dose of 375 mg / m^2^ BSA was given; the former with combination of low-dose cyclosporine treatment followed by remission and NC was relieved again 3 months after treatment, while the latter with prednisone increased to 40 mg QD followed by remission within 2 weeks. There were no relapsed patients in the SN-1 group and the SN-2 group.

**Fig. 3:**
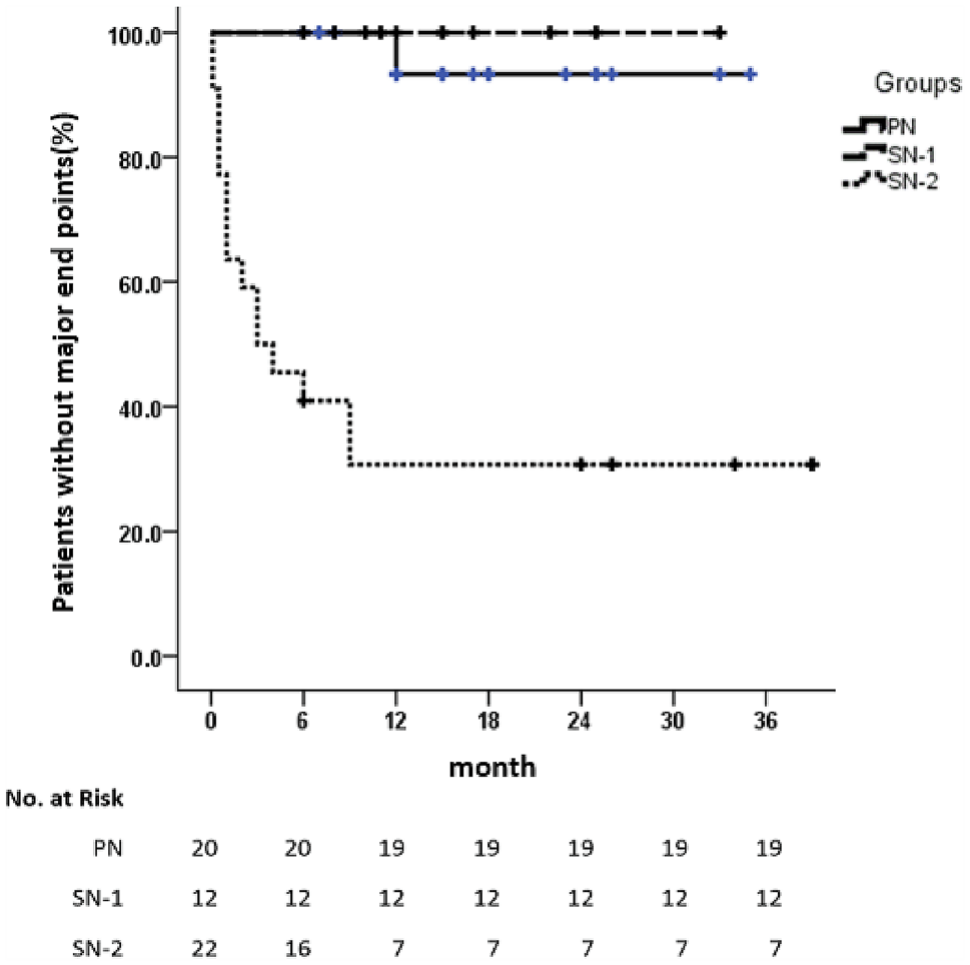
Kaplan–Meier curves for major endpoints

During the entire follow-up period, a total of two patients died, with a mortality rate of 3.33%, both of whom were in the SN-2 group. One of the two patients, with primary disease of anti-GBM combined with ANCA-associated AAV, scleroderma and heart valve disease, died of cardiac pump failure 6 months after RTX treatment. The other patient, with primary disease of AAV combined with pulmonary hemorrhage, died of pulmonary infection 3 months after RTX treatment. Sixteen patients (29.6%) reached ESRD or maintenance dialysis, including one case (5%) in the PN group, zero cases in the SN-1 group and 15 cases (68.2%) in the SN-2 group (*χ*^2^ = 25.846, *P* < 0.001). The results of Kaplan–Meier survival analysis (Fig. 3) showed a significant difference in prognosis among the three groups (*χ*^2^ = 40.150, *P* < 0.001). At the end of follow-up, 28% of patients in the SN-2 group had no endpoints, which was much lower than 95.2% in the PN group (*χ*^2^ = 26.081, *P* < 0.001) and 100% in the SN-1 group (*χ*^2^ = 15.890, *P* < 0.001). And there was no difference between the PN group and the SN-1 group (*χ*^2^ = 0.476, *P* = 0.490). The time of reaching the endpoints in the SN-2 group was 3.61 ± 2.91 months, while only one patient with FSGS in the PN group received regular dialysis 12 months after RTX treatment.

### Therapeutic safety

Of the 60 patients included, a total of 26 (43.3%) patients had 47 adverse events in addition to endpoints. Specifically, 34 patients (56.6%) had infection, eight patients (13.3%) had WBC reductions, and five patients (8.3%) had infusion reactions. Among the complications of infection, lung and respiratory tract were the most common infection sites, and bacteria were the most common pathogens. Two cases of CMV viremia (CMV-DNA positive, no symptoms of organ infection) in the PN group and 4 in the SN-2 group received antiviral therapy. Fungal infections were only seen in the SN groups, with three cases of candida albicans pneumonia in the SN-2 group and one case of Pneumocystis carinii pneumonia in each of the SN-1 group and the SN-2 group. Multiple infections (combination of bacterial, fungal and or viral infections) occurred in four cases in the SN-2 group and one case in the SN-1 group. Further statistical analysis was performed for severe infections that requiring hospitalization or a course of treatment for more than 2 weeks). The results revealed that the incidence of severe infections within 6 months of RTX treatment was highest at 50% in the SN-2 group, middle at 25% in the SN-1 group, and lowest at 10% in the PN group, with statistically significant differences among the three groups (*χ*^2^ = 8.198, *P* = 0.017). A before-and-after study revealed no significant difference in number of patients with severe infection within 6 months before and after RTX treatment (Table 4). Transient WBC reduction occurred in all the three groups; among five cases of infusion reactions, two patients showed airway spasm with palpitations and thus sopped RTX treatment, while the other three cases with mild symptoms, including two patients presenting with skin urticaria and one presenting with fever, were quickly relieved after symptomatic treatment and thus continued RTX treatment.

**Table 4:** Incidence of infection in three groups of patients

Adverse events		Groups			
	Cases	PN (*N* = 20)	SN-1 (*N* = 12)	SN-2 (*N* = 22)	
Infection occurred 6 months after RTX treatment, *N* (%)	24	9(45.5%)	4(27.3%)	11(50%)	*χ*^2^ = 0.878, *P* = 0.645
Infection occurred 6 months after RTX treatment, times (times/person-year)	34	10(1.1)	5(0.83)	19(1.72)	*H* = 3.197, *P* = 0.202
Severe infection occurred 6 months after RTX treatment, N (%)	16	2(10%)	3(25%)	11(50%)	*χ*^2^ = 8.198, *P* = 0.017
Severe infection occurred 6 months after RTX treatment, times (times/person-year)	22	3(0.3)	4(0.67)	15(1.36)	*H* = 7.496, *P* = 0.024
Severe infection occurred 6 months before RTX treatment N(%)	15	2(10%)	3(25%)	10(45.5%)	*χ*^2^ = 6.497, *P* = 0.039
Severe infection occurred 6 months before RTX treatment, times (times/person-year)	16	2 (0.2)	3(0.54)	11(1)	*H* = 6.699, *P* = 0.035
Comparison of severe infection cases 6 months before and after RTX treatment^a^	*H* = 111.0, *P* = 0.239	*H* = 6.000, *P* = 0.705	*H* = 4.000, *P* = 0.564	*H* = 22.50, *P* = 0.329	
Pulmonary infection	11	1	3	7	
Bacteria	10	1	2	7	
Fungus	5	0	1	4	
Virus	2	0	1	1	
Multiple infection	5	0	1	4	
Respiratory tract infection	8	4	2	2	
Intestinal infection	2	0	0	2	
Acute pancreatitis	1	0	0	1	
Urinary tract infection N	3	2	0	1	
Herpes zoster N	2	0	0	2	
Gingivitis	1	1	0	0	
CMV viremia	6	2	0	4	

Chi-squared test was used for the comparison of rates between groups, and Kruskal–Wallis test for independent samples was used for the comparison of measurement data between groups

^a^Wilcoxon signed rank sum test for dependent samples was used

### Results of Cox regression analysis

After correcting for gender, age, time of onset, and Upro and ALB and other parameters before RTX treatment, the level of eGFR prior to RTX treatment was a protective factor for the occurrence of the primary endpoints (HR 0.910, 95% CI 0.847–0.976), that is, every 1 ml/min increase in eGFR prior to RTX treatment would decrease the risk of primary endpoints by 9.0 percent (Table 5).

**Table 5:** Univariate and multivariate Cox regression analysis of risk factors for primary endpoints

Factor	Univariate			Multivariate		
	*HR* value	95%*CI*	*P* value	*HR* value	95%*CI*	*P* value
Age	0.993	0.965–1.002	0.639	–	–	0.243
Male	0.483	0.180−1.301	0.150	–	–	0.855
Onset time	1.004	0.998−1.010	0.172	–	–	0.346
Baseline eGFR	0.993	0.856−0.973	0.005	0.910	0.847–0.976	0.009
Baseline ALB	1.060	0.998−1.126	0.058	–	–	0.176
Baseline Upro	0.847	0.074−0.963	0.011	–	–	0.401
Baseline WBC	0.860	0.725−1.020	0.082		–	0.361
Baseline L	0.511	0.236−1.105	0.088	–	–	0.385
Baseline RBC	0.433	0.207−0.908	0.027	–	–	0.416

The variable entry method is Forward LR (forward stepwise regression based on maximum-likelihood estimation). “–” indicates the variables that are not in the regression equation (i.e., the variables with no statistical difference), and their HR value and 95% CI are not shown

*eGFR* estimated glomerular filtration rate; *Upro* urine protein; *ALB* albumin; *UA* uric acid; *WBC* white blood cell; *L* lymphocyte count; *RBC* red blood cell

## Discussions

Autoimmune glomerular diseases with RNS as the clinical manifestation have some common characteristics: insensitive to or dependent on glucocorticoids, or easy to relapse, with poor efficacy or intolerance after treatment with a variety of immunosuppressants. In addition, patients with this kind of disease are characterized by long medical history and rapid progression of renal insufficiency. RTX, as an anti-CD20 monoclonal antibody, has a mechanism different from traditional immunosuppressant, thus providing a new option for the treatment [20]. And there is a lack of randomized controlled trials (RCTs) to confirm whether the application of RTX can achieve remission and improve renal prognosis in autoimmune nephropathy manifested by RNS. All the subjects in this study were RNS patients, including PN and SN. And SN patients with eGFR < 30 ml/min using RTX as a salvage therapy was also included.

This study analyzed the efficacy and safety of RTX in the treatment of different types of RNS. The results showed that after RTX treatment, PN patients and SN-1 patients with better basal renal function had a higher remission rate and stable renal function. Only a few SN-2 patients with poor basal renal function achieved remission and most SN-2 patients progressed to ESRD or required maintenance dialysis.

In a prospective study by Xin Wang et al., all 36 IMN patients manifested as RNS, 15 (41.7%) of them achieving partial (*n* = 13) or complete (*n* = 2) remission and maintaining stable renal function after RTX treatment, whereas patients who did not respond to the treatment experienced a progressive decrease in eGFR [21]. Effectiveness of RTX in adults with MCD lacks support from randomized controlled trials. The results of an observational study by Takashi Takei et al. [10] and a retrospective study by Helene Munyentwali et al. [7] both suggested that RTX could significantly reduce relapses and glucocorticoids dosage in adult MCD patients with steroid-dependent or frequent relapses. In our study, PN group (mainly IMN and MCD patients) achieved a high remission rate, which was consistent with the findings of the previous researches. Regarding adult patients with FSGS, case review studies have shown that RTX is mostly effective in glucocorticoids-dependent RNS [9], while is poorly effective in glucocorticoids-resistant RNS [22]. In our study, there was only one patient with FSGS who presented with glucocorticoids-resistant, and the salvage treatment of RTX was ineffective and the patient quickly progressed to ESRD.

In nephropathy secondary to autoimmune diseases, AAV and SLE are common causes. Geetha's post-hoc analysis of the RAVE study [23] showed that 61% of 51 patients with AAV in the RTX group who achieved complete remission 6 months after treatment, with a remission rate of 75% in 25 relapsed cases. However, patients with advanced renal insufficiency (serum creatinine level > 4.0 mg/dl) were excluded from that study. Another RCT study by Jones [17] included 44 patients with median eGFR at 18 ml/min/1.73m^2^, and the inducing remission rate was 78% in the RTX group. With regard to LN, although the results of RCT studies were negative, observational studies have shown that RTX may be beneficial for refractory LN that has failed to respond to standard treatment. In Melander, C.'s study [16], there were a total of 20 patients with severe refractory LN (18 cases manifested as NC). Among the 20 patients, five patients had an eGFR < 30 ml/min, of which two cases achieving partial remission and three cases received maintenance hemodialysis after treatment. The other 15 patients had an eGFR > 30 ml/min, of which seven had complete remission, four had partial remission after treatment, and the remaining patients had undetailed data. In the present study, LN was predominant in both SN-1 and SN-2 groups, followed by AAV, with remission rate of 66.7% in the SN-1 group and 27.3% in the SN-2 group. Our result is similar to the findings reported in the above studies.

In terms of treatment safety, infection was the most common adverse event in our study, which is consistent with previous studies. On the whole, the incidence of infection after RTX treatment in the three groups was 1.26 times per person-year, and the severe infection rate was 0.81 times per person-year. In previous studies, limited data are available on the complications of infections in NS patients with RTX based regimens. The infection rate was 0.34 times per person year in the IMN patients (MENTOR study) [3], 0.66 times per person year in the AAV patients (RITUXVAS study) [17]. Infections occurred in 84.9% of 72 LN patients in the RTX group in Rovin’s study, including respiratory tract infections (28.8%), urinary tract infections (23.3%) and herpes zoster (15.1%) [11]. In addition, a case review analysis by Claire Trivin et al. [24] showed that the incidence of infection was 0.216 time per person year in 98 nephropathy patients treated with RTX. According to this case review analysis, the main indications for RTX therapy were membranoproliferative glomerulonephritis (mainly cryoglobulinemia-associated nephropathy), membranous nephropathy, IMN, LN and AAV. However, only 44.9% of patients in this analysis had nephropathy at the start of RTX treatment. In the case review study, prior to RTX treatment, 69 patients (70.4%) received corticosteroids and 62 patients (63.3%) had received other immunosuppressants. In our study, the incidence of infections was higher compared to the above literature. This is because the subjects included in our study were RNS patients who experienced long-term treatment of glucocorticoids and multiple immunosuppressants, with low serum albuminemie and malutrition. Therefore, this population is at high risk of infection. Besides, severe infections did not increase significantly 6 months after RTX treatment compared to the baseline, suggesting that RTX treatment does not increase the incidence of infectious complications in this population, which is consistent with the results of most previous studies [2, 3, 5, 11, 17]. However, it should be noted that there was an increase in cases of multiple infections and opportunistic infections (CMV viremia, fungal infections) after RTX treatment in our study. Herpes zoster virus, CMV, and PJP infections after RTX treatment have also been reported in previous studies [24–28], suggesting that RTX treatment further inhibits humoral immunity to increase the risk of infection.

In addition, in this study, the SN-2 group had a significantly higher rate of severe infections after RTX treatment than the other two groups. Claire Trivin’s study have shown that eGFR reduction is associated with a high risk of infection [24]. This is consistent with our research results.

This study is a single-center observational study with a small sample size, thus causing the following limitations. First, the PN group has a relatively high basic eGFR level and lacks a subgroup with eGFR < 30 ml/min. Therefore, our study cannot reflect the efficacy of RTX for PN patients with a low basic eGFR. Second, in this study, the PN group is mainly IMN, while the SN group is mostly LN, indicating an uneven distribution of disease composition and poor representativeness. Collectively, the results observed in this study cannot be blindly applied in other RNS populations.

## Conclusion

The application of RTX in RNS patients with eGFR ≥ 30 ml/min/m^2^ (including PN and SN) can effectively reduce urine protein, increase plasma ALB levels, and has no effect on eGFR. But the application of RTX in secondary RNS patients with eGFR < 30 ml/min/m^2^ has little benefit and a high incidence of adverse events, especially severe infectious complications.
